# The cardiac calsequestrin gene transcription is modulated at the promoter by NFAT and MEF-2 transcription factors

**DOI:** 10.1371/journal.pone.0184724

**Published:** 2017-09-08

**Authors:** Rafael Estrada-Avilés, Gabriela Rodríguez, Angel Zarain-Herzberg

**Affiliations:** Department of Biochemistry, School of Medicine, National Autonomous University of Mexico, Mexico City, Mexico; Newcastle University, AUSTRALIA

## Abstract

Calsequestrin-2 (CASQ2) is the main Ca^2+^-binding protein inside the sarcoplasmic reticulum of cardiomyocytes. Previously, we demonstrated that MEF-2 and SRF binding sites within the human *CASQ2* gene (*hCASQ2*) promoter region are functional in neonatal cardiomyocytes. In this work, we investigated if the calcineurin/NFAT pathway regulates *hCASQ2* expression in neonatal cardiomyocytes. The inhibition of NFAT dephosphorylation with CsA or INCA-6, reduced both the luciferase activity of *hCASQ2* promoter constructs (-3102/+176 bp and -288/+176 bp) and the CASQ2 mRNA levels in neonatal rat cardiomyocytes. Additionally, NFATc1 and NFATc3 over-expressing neonatal cardiomyocytes showed a 2-3-fold increase in luciferase activity of both *hCASQ2* promoter constructs, which was prevented by CsA treatment. Site-directed mutagenesis of the -133 bp MEF-2 binding site prevented trans-activation of *hCASQ2* promoter constructs induced by NFAT overexpression. Chromatin Immunoprecipitation (ChIP) assays revealed NFAT and MEF-2 enrichment within the -288 bp to +76 bp of the *hCASQ2* gene promoter. Besides, a direct interaction between NFAT and MEF-2 proteins was demonstrated by protein co-immunoprecipitation experiments. Taken together, these data demonstrate that NFAT interacts with MEF-2 bound to the -133 bp binding site at the *hCASQ2* gene promoter. In conclusion, in this work, we demonstrate that the Ca^2+^-calcineurin/NFAT pathway modulates the transcription of the *hCASQ2* gene in neonatal cardiomyocytes.

## Introduction

In cardiomyocytes, the sarcoplasmic reticulum (SR) is the main intracellular Ca^2+^ reservoir. The SR has a main role in the Ca^2+^ homeostasis control of cardiomyocytes [1]. The SR is a complex network of membranous structures constituted by longitudinal tubules interconnected by wide cisterns. Inside the SR there are Ca^2+^ binding proteins that play fundamental roles in the Ca^2+^ homeostasis [2, 3]. The concerted action of the Ca^2+^-binding protein calsequestrin (CASQ), the Ca^2+^-release channel (RyR) and the Ca^2+^-ATPase pump (SERCA) control storage, release, and re-uptake of Ca^2+^, respectively, regulating contraction and Ca^2+^ homeostasis in skeletal and cardiac muscles [1, 4, 5].

In mammals, there are two calsequestrin isoforms encoded by two different genes. The calsequestrin-1 isoform (CASQ1) is encoded by the *CASQ1* gene (1q21) and is expressed exclusively in fast and slow-twitch skeletal muscles. The calsequestrin-2 (CASQ2) isoform is encoded by the *CASQ2* gene (1p13.3-p11), which is mainly expressed in cardiomyocytes and lesser extent in slow-twitch skeletal muscle [6–8]. The CASQ2 isoform is the main Ca^2+^ binding protein inside the terminal cisternae of SR of the cardiomyocytes [1]. CASQ2 (MW 40 kDa) is a highly acidic protein, mainly in its C-terminal region. Because of this, CASQ2 has a high Ca^2+^-binding capacity (40–50 mol Ca^2+^/mol protein) [9]. Inside the SR, CASQ2 acts as Ca^2+^ buffer, maintaining free Ca^2+^ about 1 mM [10, 11]. It is estimated that CASQ2 binds between 50–75% of total Ca^2+^ inside the SR of cardiomyocytes [12]. It has been proposed that CASQ can regulate the SR Ca^2+^ release during excitation-contraction coupling in skeletal and cardiac myocytes [13]. Inside the SR, at physiological Ca^2+^ concentration, CASQ monomers have a thioredoxin-like structure and interact with each other forming a linear polymer. The CASQ polymer forms a structure that creates a kind of matrix. Apertures inside this CASQ matrix form a channel that conducts Ca^2+^ to the RyR Ca^2+^ release channel [14]. CASQ polymer forms a multiprotein complex with the transmembrane proteins triadin (TRD) and junctin (JNC) [15]. In cardiac muscle, during the Ca^2+^-induced Ca^2+^-release, the SR Ca^2+^ levels decrease by 40–60%. When this happens, the CASQ2-TRD-JNC complex inhibits the RyR2 channel. Therefore, the activity of CASQ2-TRD-JNC-RyR2 complex helps to maintain the SR free Ca^2+^ level at 1 mM despite total SR [Ca^2+^] changes significantly between contraction cycles [12].

The first 288 bp of the human *CASQ2* gene (*hCASQ2*) promoter are highly conserved among species (100% chimpanzee, 95% mice, 98% rat, 87% chicken compared with the human). This conserved region contains a TATA-box and binding sites for MEF-2 (Myocyte Enhancer Factor-2) and SRF (Serum Response Factor) transcription factors. We have previously demonstrated that MEF-2 and SRF binding sites within this region are functional in neonatal cardiomyocytes [16]. However, it is unknown if MEF-2 and SRF are the only factors involved in the expression of *hCASQ2* gene. The *hCASQ2* gene expression is poorly understood. Just a few reports concerning the regulation of *hCASQ2* gene expression are found in the literature [6, 8, 16–18].

The calcineurin/NFAT pathway is functional in cardiomyocytes and regulates gene expression mediated by intracellular calcium concentration ([Ca^2+^]) changes [19]. The NFAT (nuclear factor of activated T-cells) transcription factor regulates the expression of muscle specific proteins, such the β-myosin heavy chain (β-MHC), skeletal muscle myosin and smooth muscle myosin heavy chain [20–22]. Acting together or separately, MEF-2 and NFAT regulate the expression of important genes such as β-MHC, Nur77, and BNP [20, 23–28]. It is known that MEF-2 and NFAT can interact with each other and regulate Ca^2+^-dependent gene expression in T lymphocytes [26]. Besides, it has been reported that an interaction between NFAT and SRF can regulate the expression of the α-actin gene in smooth muscle cells [29].

In this work, we investigated the role of calcineurin/NFAT pathway in the regulation of the *hCASQ2* gene expression. We demonstrated that the inhibition of calcineurin/NFAT pathway reduced the *hCASQ2* gene transcription in primary cultures of neonatal rat cardiomyocytes. Likewise, we demonstrated that NFAT overexpression induced transcription of the *hCASQ2* gene. Our experiments also showed that both MEF-2 and NFAT transcription factors are present in the *CASQ2* gene proximal promoter and they physically interact with each other in neonatal cardiomyocytes.

## Materials and methods

### Cell culture

Primary cultures of cardiomyocytes were prepared from neonatal rat hearts (1–2 days-old) according to the method previously described [30]. This study has been specifically approved by the ethics committee Institutional Care and Use Committee (IACUC) of the School of Medicine, National Autonomous University of Mexico. Cells were grown in DMEM (Dulbecco´s modified Eagle´s medium from Invitrogen, CA, USA) supplemented with 10% bovine fetal serum (Gibco, MA, USA), kanamycin (60 mg/mL) (Sigma-Aldrich, MO, USA), penicillin (10 U/mL) (Gibco, MA, USA), streptomycin (10 mg/mL) (Gibco, MA, USA), amphotericin B (0.025 mg/mL) (Gibco, MA, USA) and nystatin (10 U/mL) (Sigma-Aldrich, MO, USA).

### Functional assays

Primary cultures of neonatal rat cardiomyocytes were used for transfection experiments. The plasmids containing the promoter region of the *hCASQ2* gene were cloned previously by our group in pGL3-basic (Promega, WI, USA) [16]. Two constructs of the *hCASQ2* gene were used for transfection experiments. The long *hCASQ2* gene promoter construct contains 3102 bp of the *hCASQ2* gene promoter and 176 bp of the 5´-untranslated region of exon one (pGL3-*hCASQ2*prom/Luc -3102/+176 bp). The short construct contains 288 bp of the promoter region and 176 bp of the 5´untranslated region (pGL3-*hCASQ2*prom/Luc -288/+176 bp) [16]. The expression plasmids for NFATc1 (pCR4-huNFATc1nuc, plasmid #23988) and NFATc3 (pBS-mNFATc3 EE, plasmid #17868) were purchased from Addgene (Cambridge MA, USA). Cultures were transfected with 0.5 μg of *hCASQ2* gene constructs and treated with cyclosporine A (CsA) (Sigma-Aldrich, MO, USA) for 12 h or with INCA-6 (NFAT Activation Inhibitor III, catalog number 480403, Calbiochem-Merck, Darmstadt, Germany) during 16 h. After the treatment had been completed, the luciferase activity was measured using a Wallac Victor^2^ 1420 Multilabel Counter (Perkin-Elmer, MA, USA) and the Dual-Luciferase Reporter Assay System (Promega, WI, USA). Firefly luciferase activity was normalized using *Renilla* luciferase activity or with protein concentration. For NFAT overexpression assays, 0.5 μg of the two pGL3-*hCASQ2*prom/Luc chimeric constructs, 0.025 μg of the *Renilla* luciferase plasmid pRL-CMV, and 0.5 μg of NFATc1 (pCR4-huNFATc1nuc) or NFATc3 (pBS-mNFATc3EE) expression vectors were transiently co-transfected into neonatal rat cardiomyocytes plated in 24-well plates using Lipofectamine 2000 reagent (Invitrogen, CA, USA). 24 h later, the cells were harvested, and the luciferase activity was determined using the Dual-Luciferase Reporter Assay System (Promega, WI, USA) in a Wallac Victor^2^ 1420 Multilabel Counter (Perkin-Elmer, MA, USA). Firefly luciferase activity was normalized using *Renilla* luciferase activity and protein concentration.

### Total RNA extraction and qRT-PCR

Primary cultures of neonatal rat cardiomyocytes were treated with CsA during 12 h or with INCA-6 during 30 h. Total RNA extraction was made using the TRIzol reagent (Invitrogen, CA, USA). Then 1 μg of total RNA was reversed transcribed using the SuperScript III First-Strand Synthesis Supermix (Invitrogen, CA, USA). For real-time qPCR, 4.6 μL of a 1:8 dilution of cDNA and the SYBR GreenER qPCR Supermix (Invitrogen, CA, USA) were used. The final reaction volume was 10 μL. GAPDH mRNA was used as load control. CASQ2 mRNA levels were analyzed by the method previously described [31]. CASQ2 and GAPDH primers used for qPCR are listed in S1 Table.

### Site-directed mutagenesis

The site-directed mutagenesis of the putative binding sites for MEF-2 at -133 bp, SRF at -103 bp and NFAT at -230 bp was done as follows. Briefly, 200 ng of the short plasmid construct (pGL3-*hCASQ2*prom/Luc -288/+176 bp) were subjected to a standard mutagenic PCR reaction with *Pfu* Turbo DNA polymerase (Thermo Fisher Scientific, MA, USA) and 125 ng of specific primers. The primers used for site-directed mutagenesis are listed in S1 Table. The mutagenic PCR reaction parameters were as follows: 95°C for 5 min, 18 cycles (95°C for 50 sec, 75°C for 50 sec, 68°C for 5 min) and 68°C for 7 min. The final reaction volume was 50 μL. The reaction product was digested with 10 U of methylation-sensitive enzyme *DpnI* at 37°C during 2 h. (New England Biolabs, MA, USA). *E*. *coli* DH5-αcompetent cells were transformed with the amplified products. Finally, the plasmids were purified using the PureLink plasmid DNA purification kit (Catalog K2100-04, Invitrogen, CA, USA). The mutated plasmids were used in functional assays.

### Chromatin immunoprecipitation (ChIP) assays

Primary cultures of neonatal rat cardiomyocytes were used for ChIP experiments. The cross-linking reaction was done using 1% formaldehyde for 15 min. The reaction was stopped with glycine (Sigma-Aldrich, MO, USA) at a final concentration of 0.125 M. Culture medium was removed, and the cells were washed with PBS 1X with PMSF 1 mM. The cells were then lysed with lysis buffer (Tris-HCl 50 mM pH 8.0, EDTA 10 mM, SDS 1%, and Sigma Fast Protease Inhibitor Cocktail [Sigma-Aldrich, MO, USA]). The cells were subjected to 5 sonication cycles of 15 sec ON with 90 sec OFF in a Biorruptor Pico (Diagenode, NJ, USA) sonication device. Immunoprecipitation was done with One-Day ChIP Kit (Catalog number C01010081, Diagenode, NJ, USA) following the manufacturer instructions. Sonicated chromatin was incubated with 8 μg of antibody against MEF-2c (C-21X sc313x, Santa Cruz Biotechnology Inc., CA, USA), NFATc3 (M75X sc8321, Santa Cruz Biotechnology Inc., CA, USA) or Sp1 (PEP-2 sc59x, Santa Cruz Biotechnology Inc., CA, USA) as a negative control. The primers used for PCR reaction are listed in S1 Table. The PCR reaction cycles were as follows: 10 min at 95°C, 40 cycles (30 sec at 95°C, 30 sec at 60°C, 30 sec at 72°C) and 5 min at 72°C. The final reaction volume was 20 μL.

### Western blot analysis

Primary cultures of neonatal rat cardiomyocytes were lysed with RIPA buffer (Tris-HCl 50 mM pH 7.5, NaCl 150 mM, sodium deoxycholate 1%, Triton X-100 1%, NP-40 1%, EDTA 0.3 mM pH 8.0, PMSF 1 mM, SigmaFast Protease inhibitor Cocktail 1X). Protein concentration was determined by a Bradford-based method using the Bio-Rad protein assay dye reagent (Catalog 500–0006, Bio-Rad CA, USA). The samples absorbance was measured in a Wallac Victor^2^ 1420 Multilabel Counter (Perkin-Elmer, MA, USA). Protein lysates (30 μg) were separated on 12% polyacrylamide gels and transferred to a PVDF membrane (Bio-Rad CA, USA). Membranes were blocked with 5% non-fat dry milk (Bio-Rad CA, USA) dissolved in Tris-buffered saline with 0.1% Tween (TBST 0.1%). Membranes were incubated overnight at 4°C with primary antibody against NFATc3 (M75X sc8321, Santa Cruz Biotechnology Inc., CA, USA), MEF-2c (C-21X sc313x, Santa Cruz Biotechnology Inc., CA, USA), CASQ2 (EPR4227 ab 108289, Abcam, Cambridge, UK) or β-actin (sc-130300, Santa Cruz Biotechnology Inc., CA, USA). Anti-NFAT was diluted to 2 μg/mL, anti-MEF-2 was diluted to 2μg/750 μL, anti-CASQ2 was diluted to 1:10,000 and anti-β-actin was diluted 1 to 5000. Membranes were incubated with the anti-rabbit secondary antibody (1:5000) (ThermoFisher MA, USA) for 1 h at room temperature. Signals were detected with SuperSignal West Dura reagent (34075, ThermoFisher MA, USA) in a C-Digit Blot Scanner (LI-COR, NE, USA) and analyzed using the Image-Studio Lite 5.2.5 software (LI-COR, NE, USA).

### Protein Co-immunoprecipitation assays

Protein extracts of neonatal rat cardiomyocytes were prepared with RIPA buffer. Protein extracts (1 mg) were immunoprecipitated overnight at 4°C with 5 μg of anti-NFAT (ab 2722, Abcam, Cambridge, UK) or 5 μg of anti-MEF-2c (C-21X sc313x, Santa Cruz Biotechnology Inc., CA, USA) antibodies. Immunoprecipitated proteins were recovered using Protein A Sepharose CL-4B (17-0780-01, GE Health Care, IL, USA). Immunoprecipitated proteins were analyzed by Western blot with anti-MEF-2c (C-21X sc313x, Santa Cruz Biotechnology Inc., CA, USA) antibody.

### Sequence analysis

The sequence analysis was made using MacVector 6.5.3 (Accelrys) and BLAST tool (NCBI), and MathInspector (Genomatix).

### Statistical analysis

Values are expressed as the mean of three independent experiments +/- SEM (standard error). Data were analyzed using GraphPad Prism 5 (GraphPad Software Inc, CA, USA) using ANOVA and multiple comparison tests of Bonferroni or Dunnet. P values < 0.05 were considered as statistical significant.

## Results

### Inhibition of the calcineurin/NFAT pathway decreases CASQ2 mRNA synthesis in neonatal cardiomyocytes

**Fig 1:**
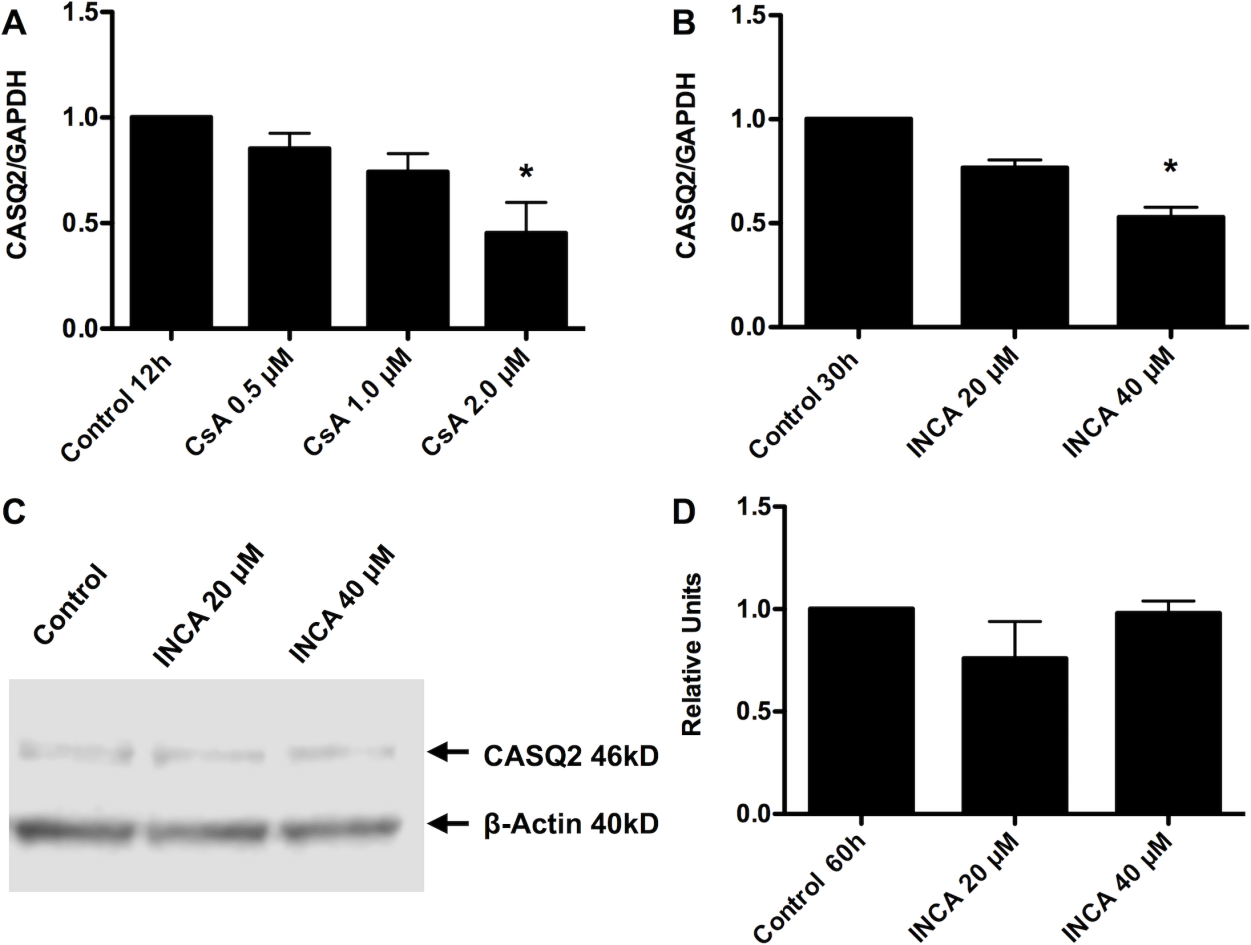
Inhibition of NFAT activity with CsA and INCA-6 decreases CASQ2 mRNA synthesis in neonatal cardiomyocytes. **A)** Neonatal rat cardiomyocytes were treated with DMSO as the vehicle (Control) or CsA for 12 h. The cells were then harvested and CASQ2 mRNA was determined by real-time PCR. GAPDH mRNA was used as normalizing gene **B)** Neonatal rat cardiomyocytes were treated with vehicle (Control) or INCA-6 for 30 h. The cells were then harvested, and CASQ2 mRNA was determined by real-time PCR. GAPDH mRNA was used as normalizing gene. Results of RT-qPCR data are expressed as the mean of three independent experiments +/- SEM. *p values < 0.05 *vs* control. **C)** Neonatal rat cardiomyocytes were treated with INCA-6 for 60 h. The cells were then harvested and CASQ2 protein levels were analyzed by Western blot. β-actin protein levels were used for normalization. A picture of a representative the gel is shown. **D)** Densitometry analysis of representative experiments (*n* = 2) was made using the Image-Studio Lite 5.2.5 software, LI-COR, NE, USA. (The data showed in the graphs can be found as supporting information in the S1 Dataset file. The complete image of the blot can be found in S1 Fig file).

In order to verify if the inhibition of calcineurin/NFAT pathway reduces the CASQ2 expression in our experimental conditions, we treated neonatal rat cardiomyocytes in culture with CsA during 12 h. The results showed that the inhibition of calcineurin-mediated NFAT activation with CsA decreased the CASQ2 mRNA up to 50% **(Fig 1A)**. Besides NFAT, calcineurin has other molecular targets [32]. Therefore, we used the NFAT activation-inhibitor INCA-6, which specifically inhibits the interaction between calcineurin and NFAT. Thus, INCA-6 inhibits calcineurin/NFAT pathway activation without affecting the other calcineurin targets [33]. The inhibition of calcineurin/NFAT pathway activation with INCA-6 also decreased the CASQ2 endogenous mRNA levels **(Fig 1B)**. However, the inhibition of calcineurin/NFAT pathway, with INCA-6, did not produce a significant reduction in CASQ2 protein levels **(Fig 1C and 1D)**. We presume that the long half-life of CASQ2 did not allow us to see a significant reduction in the protein levels.

Nevertheless, the results of CASQ2 mRNA quantification demonstrate that calcineurin/NFAT pathway might have a role in *CASQ2* gene transcription regulation.

### Inhibition of NFAT activation reduced transcriptional activity of *hCASQ2* gene constructs

**Fig 2:**
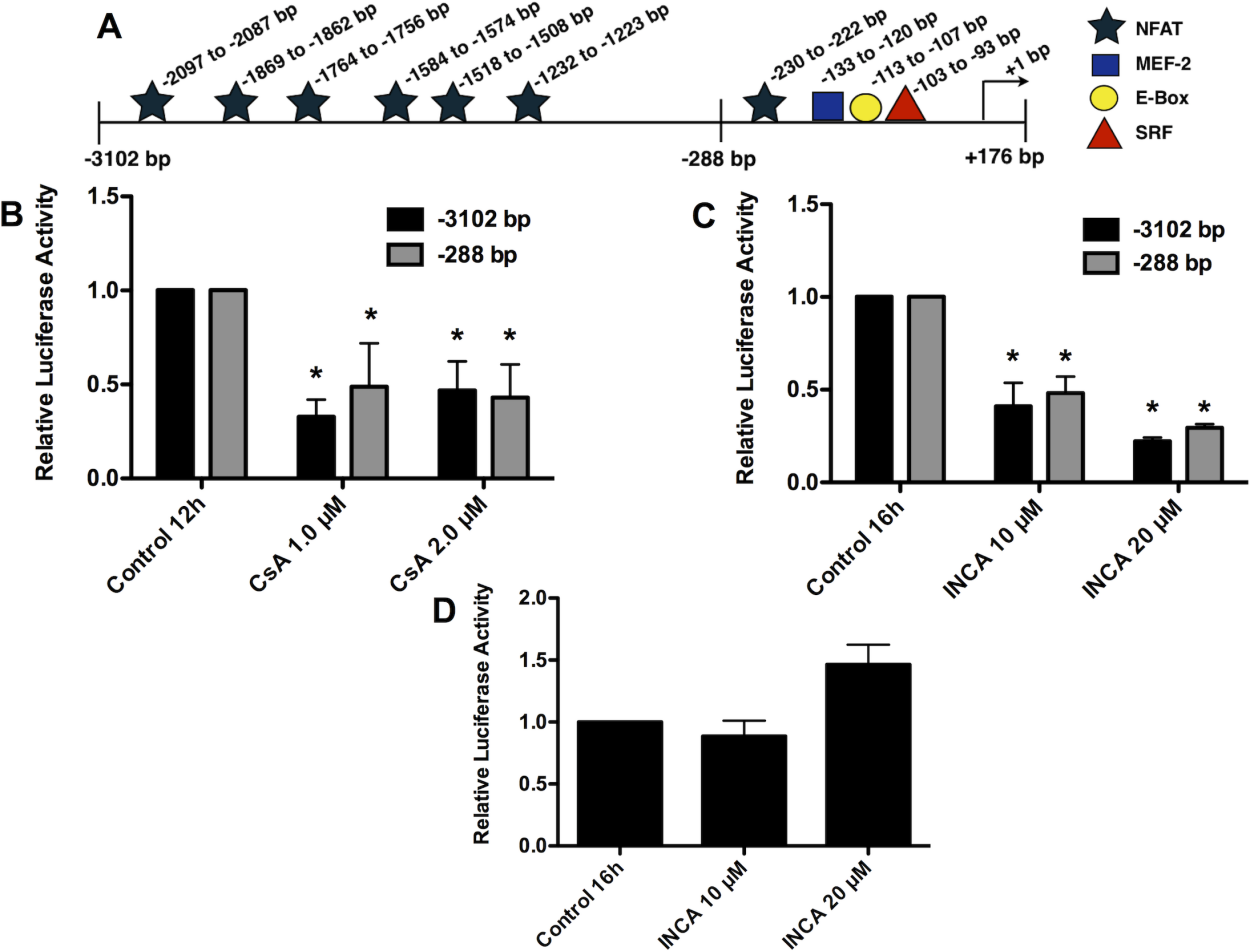
Inhibition of NFAT activity with CsA and INCA-6 reduced the transcriptional activity of the *hCASQ2* promoter constructs. The two *hCASQ2* gene chimeric constructs were transfected into neonatal rat cardiomyocytes cultures. **A)** Diagram of the *hCASQ2* gene promoter constructs. The boundaries of the two *hCASQ2* gene chimeric constructs (-3102/+176 bp and -288/+176 bp) are indicated. As well, the relative locations of potential binding sites for transcription factors found by sequence analysis are shown. **B)** Luciferase activity of neonatal cardiomyocytes transfected with the pGL3-*hCASQ2*prom/Luc -3102/+176 bp construct (black bars) or the pGL3-*hCASQ2*prom/Luc -288/+176 bp construct (gray bars) and treated with vehicle (Control) or with CsA for 12 h. **C)** Luciferase activity of neonatal cardiomyocytes transfected with the pGL3-*hCASQ2*prom/Luc -3102/+176 bp (black bars) or the pGL3-*hCASQ2*prom/Luc -288/+176 bp construct (gray bars) and treated with vehicle (Control) or INCA-6 for 16 h. **D)** Luciferase activity of neonatal cardiomyocytes transfected with the pGL3-promoter construct, containing the SV-40 promoter (Promega) and treated with vehicle or INCA-6 for 16 h. Results are expressed as the mean of three independent experiments +/- SEM. The luciferase activity of the control condition is given the arbitrary value of 1. *p values <0.05 *vs* control. (The data showed in the graphs can be found as supporting information in the S1 Dataset file).

Once we determined that the inhibition of NFAT activation with INCA-6 and CsA decreases CASQ2 mRNA synthesis in neonatal cardiomyocytes, we focused on elucidating the mechanism responsible for this effect. *In silico* DNA sequence analysis revealed seven potential NFAT binding sites in the *hCASQ2* gene promoter **(Fig 2A)**. Therefore, we decided to investigate if the inhibition of *CASQ2* mRNA synthesis induced by calcineurin inhibitors INCA-6 and CsA was transcriptionally mediated. For this purpose, we performed functional assays with two chimeric constructs (pGL3-*hCASQ2*prom/Luc -3102/+176 bp and pGL3-*hCASQ2*prom/Luc -288/+176 bp) of the *hCASQ2* gene promoter previously cloned by our group in the pGL3-basic vector [16]. Both constructs were transfected into neonatal rat cardiomyocytes, then the cells were treated with CsA or with INCA-6. In agreement with the CASQ2 mRNA quantification results, both inhibitors (CsA and INCA-6) reduced the transcriptional activity of both pGL3-*hCASQ2*prom/Luc chimeric constructs but had not the same effect on the pGL3-promoter construct which contains the SV-40 promoter **(Fig 2B–2D)**. Accordingly, the reduction of CASQ2 mRNA synthesis induced by the inhibition of the calcineurin/NFAT pathway is transcriptionally mediated.

### Over-expression of NFATc1 and NFATc3 increased transcriptional activity of *hCASQ2* gene constructs

**Fig 3:**
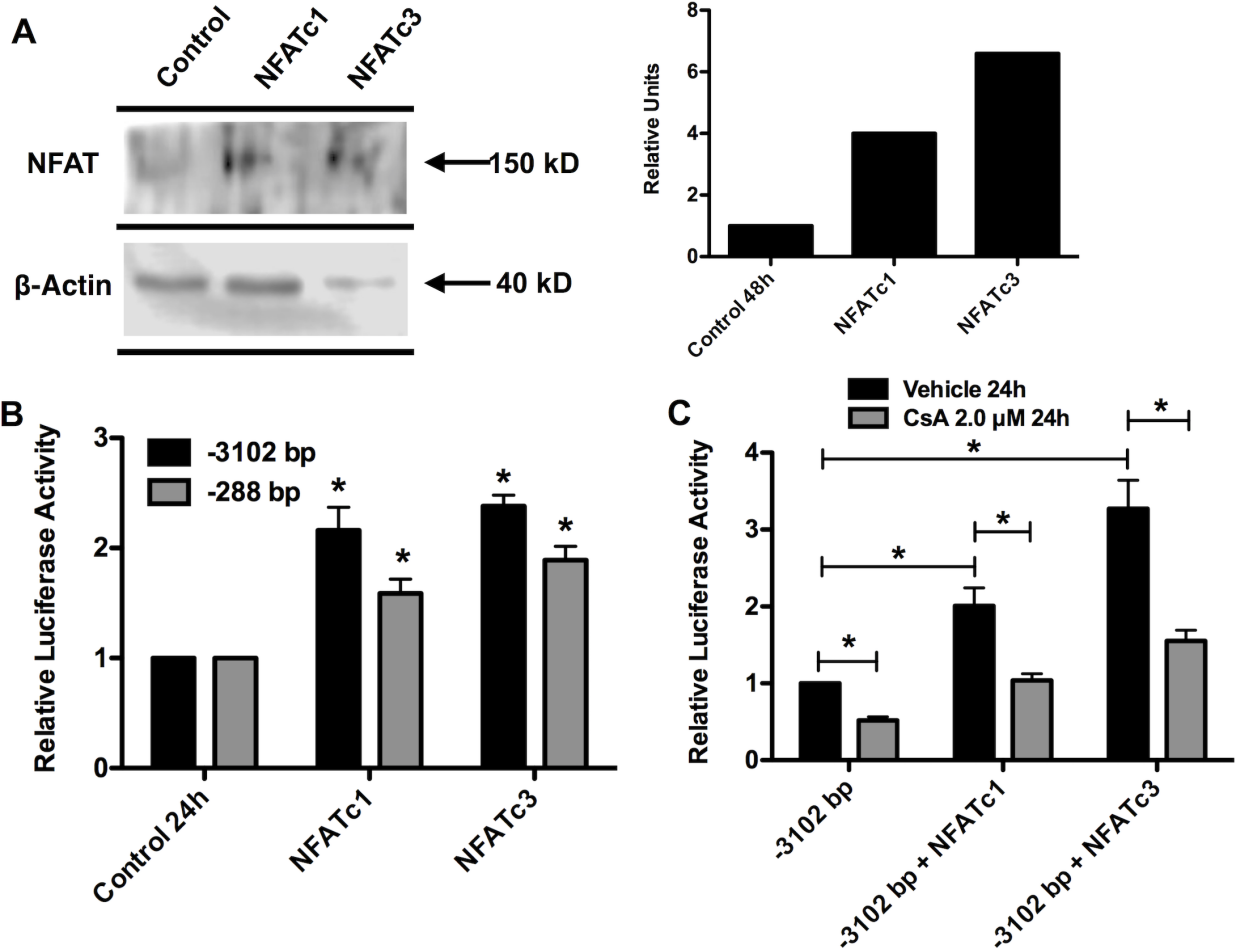
Overexpression of NFAT increased transcriptional activity of *hCASQ2* gene constructs. **A)** To verify the NFAT overexpression, the NFATc1 or NFATc3 expression vectors were transfected into neonatal rat cardiomyocytes. After 48 h, the cells were harvested and NFATc1 and NFATc3 protein levels were evaluated by Western blot. A picture of a representative gel is shown. Densitometry analysis of the blot was made using the Image-Studio Lite 5.2.5 software, LI-COR, NE, USA. **B)** Luciferase activity of neonatal cardiomyocytes co-transfected with pGL3-*hCASQ2*prom/Luc -3102/+176 bp (black bars) or pGL3-*hCASQ2*prom/Luc -288/+176 bp (gray bars) and the NFATc1 or NFATc3 expression vectors. After 24 h, the cells were harvested and luciferase activity was measured. **C)** Luciferase activity of neonatal cardiomyocytes co-transfected with pGL3-*hCASQ2*prom/Luc -3102/+176 bp construct and NFATc1 or NFATc3 expression vectors as above. Then, cells were treated with vehicle (black bars) or CsA (gray bars). After 24 h, the cells were harvested, and luciferase activity was measured. Results for functional assays (Fig 3B and 3C) are expressed as the mean of three independent experiments +/- SEM. The luciferase activity of control condition is given the arbitrary value of 1. *p values <0.05 were considered statistically significant. (The data showed in the graphs can be found as supporting information in the S1 Dataset file. The complete image of the blot can be found in S2 Fig file).

To further characterize the role of NFAT in the transcription of the *hCASQ2* gene, we overexpressed NFATc1 and NFATc3 proteins in neonatal cardiomyocytes and verified the NFAT overexpression by Western blot **(Fig 3A)**. We evaluated the effect of NFAT overexpression on the transcriptional activity of both *hCASQ2* gene promoter constructs by co-transfecting the long and short pGL3-*hCASQ2*prom/Luc chimeric constructs with the NFATc1 or NFATc3 expression vectors. The results showed that either NFATc1 or NFATc3 overexpression induces the transcriptional activity of both promoter constructs **(Fig 3B)**. Additionally, the CsA treatment together with the overexpression of NFATc1 or NFATc3 prevented the transactivation effect induced by NFAT overexpression on *hCASQ2* gene promoter long construct **(Fig 3C).** Because both *hCASQ2* gene promoter constructs responded in a similar way to the calcineurin/NFAT pathway inhibition and the NFAT overexpression **(Figs 2 and 3)**, we concluded that the response element responsible for this effect must be located within the short construct. This construction contains the region between -288 bp to +176 bp of the *hCASQ2* gene promoter. Because the sequence analysis of *hCASQ2* gene promoter revealed a potential NFAT binding site within this region, we investigated the functionality of this NFAT site.

### The -133 bp MEF-2 binding site is responsible for NFAT-induced transactivation of the *hCASQ2* gene promoter

**Fig 4:**
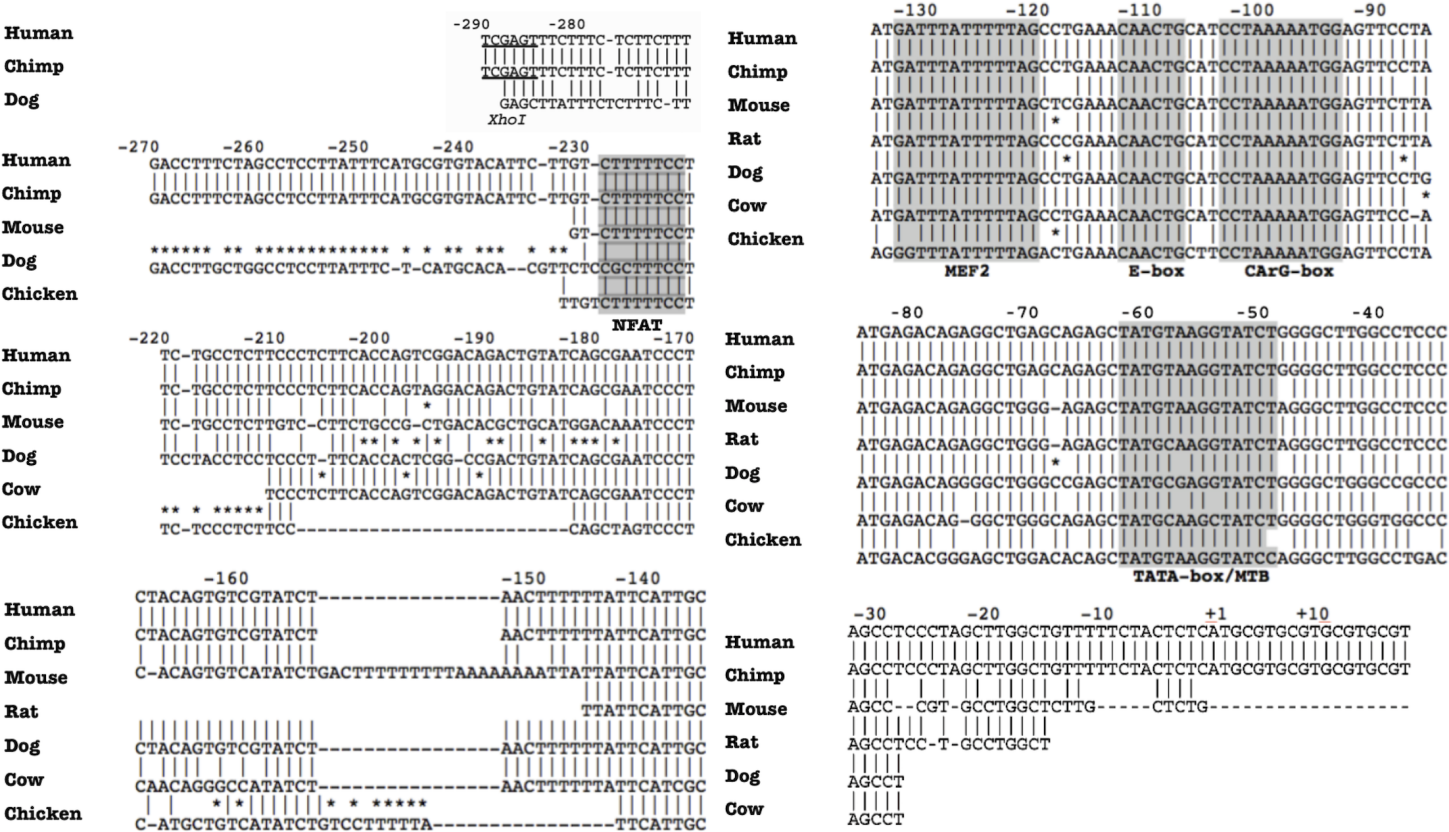
Sequence analysis of *hCASQ2* gene promoter. The sequence analysis was made using MacVector 6.5.3 and BLAST tool from NCBI. The 5´-regulatory region is highly conserved among species. The analysis revealed a potential binding site for NFAT located between -230 to -222 bp, which is conserved among species. Within this region, there are also the binding sites for MEF-2 and SRF transcription factors between -130 to -120 bp and -103-93 bp, respectively (Modified from Reyes-Juarez JL. et al. 2007).

**Fig 5:**
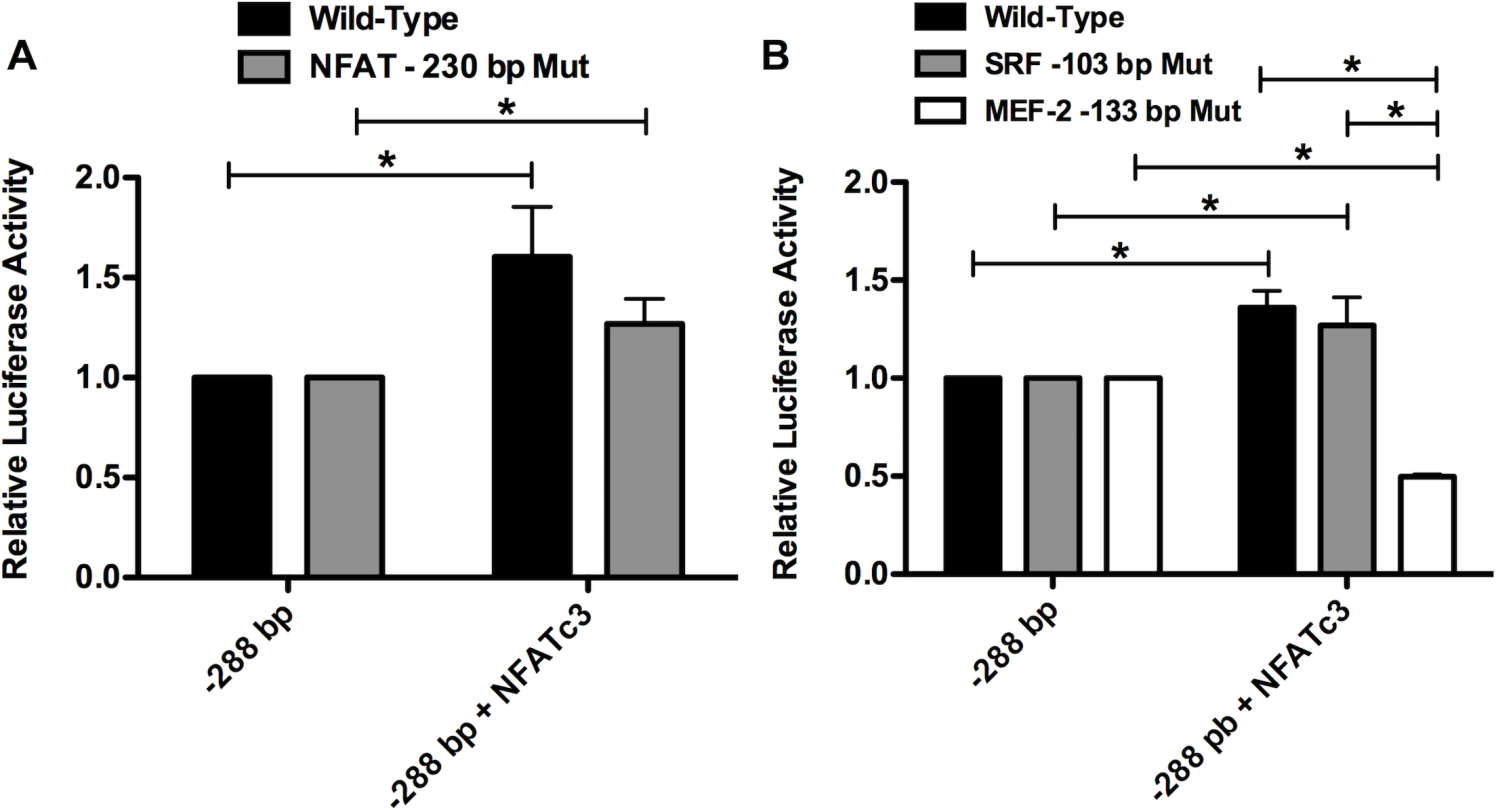
The NFAT-induced transactivation of *hCASQ2* gene promoter is mediated by the -133 bp MEF-2 binding site. **A)** Luciferase activity of neonatal cardiomyocytes co-transfected with the pGL3-*hCASQ2*prom/Luc -288/+176 bp wild-type construct (black bars) or the -230 bp NFAT site mutated construct (gray bars) and the NFATc3 expression vector. **B)** Luciferase activity of neonatal cardiomyocytes co-transfected with pGL3-*hCASQ2*prom/Luc -288/+176 bp wild-type construct (black bars), the -103 bp SRF site mutated construct (gray bars) or the -133 bp MEF-2 site mutated construct (white bars) and the NFATc3 expression vector. After 24 h, the cells were harvested, and luciferase activity was determined. Results are expressed as the mean of three independent experiments +/- SEM. The -288 bp construct luciferase activity is given the arbitrary value of 1. *p values <0.05 were considered statistically significant. (The data showed in the graphs can be found as supporting information in the S1 Dataset file).

The results of the functional assays suggest that NFAT transcription factor is involved in *hCASQ2* gene transcription regulation in neonatal cardiomyocytes. As mentioned above, *in silico* DNA sequence analysis of the *hCASQ2* gene promoter revealed a putative NFAT binding site (-230 5´-GTCTTTTTCC-3´-222) that is conserved among species **(Fig 4)**. To find out if this putative NFAT binding site is functional, we performed site-directed mutagenesis of this putative NFAT site and evaluated the effect on the transcriptional activity of the pGL3-*hCASQ2*-Luc -288/+176 bp construct by functional assays. The results showed that the mutagenesis of the putative -230 bp NFAT site had no effect on the transcriptional activity of the proximal *hCASQ2* promoter construct, suggesting that there is not a direct interaction of NFAT with the putative NFAT element located in this region **(Fig 5A)**. We have previously demonstrated that MEF-2c binds to the *-*133 bp *hCASQ2* site located within the promoter region and activates transcription of the gene in neonatal cardiomyocytes [16]. We have also demonstrated that the -103 bp SRF site of the *hCASQ2* gene is functional in neonatal cardiomyocytes [16]. Therefore, in this study, we investigated the possibility that NFAT may interact with MEF-2 at the -133 bp site or with the SRF that binds at position -103 bp. To examine this hypothesis, we generate mutations of the -133 bp MEF-2 and the -103 SRF sites in the short *hCASQ2* gene construct. We performed functional assays using these *hCASQ2* mutated constructs. The results showed that the mutated -103 bp SRF construct was still trans-activated by NFAT overexpression **(Fig 5B)**. On the other hand, we found that the -133 bp MEF-2 mutated construct was no longer trans-activated by NFAT overexpression, suggesting that NFAT may directly interact with MEF-2 bound to the proximal MEF-2 site at -133 bp **(Fig 5B)**.

### A direct interaction between MEF-2c and NFATc3 transcription factors regulate expression of the *CASQ2* gene

**Fig 6:**
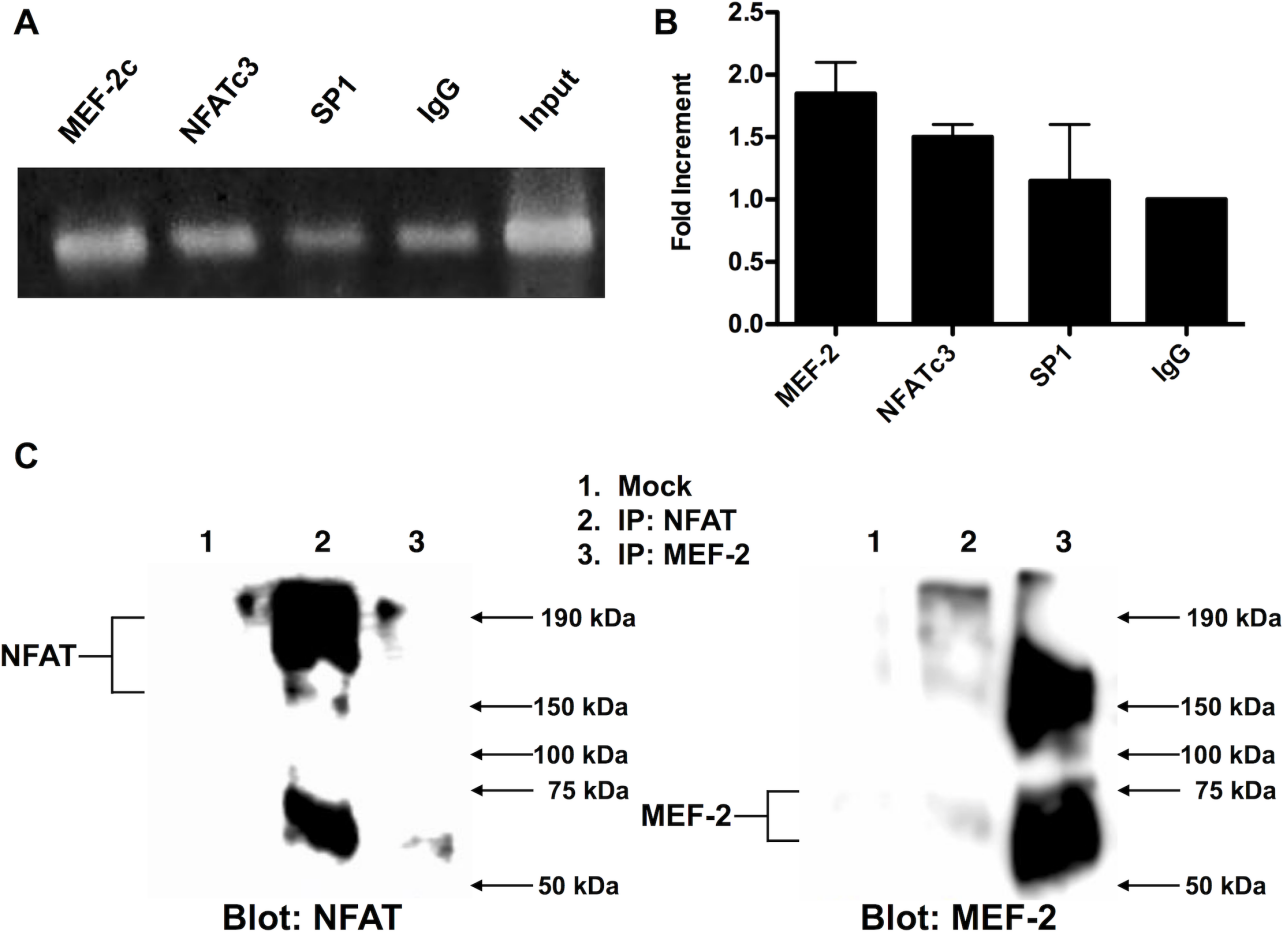
MEF-2 and NFAT transcription factors bind to the *CASQ2* gene promoter and interact with each other. **A)** Chromatin Immunoprecipitation (ChIP) assay of the rat *CASQ2* gene promoter; fragmented chromatin was incubated with antibodies against MEF-2c, NFATc3, Sp1 and IgG. PCR was performed to amplify the region between -259 to -21 bp of *CASQ2* gene promoter. A picture of a representative gel is shown. **B)** Densitometry analysis of representative experiments (*n* = 2) was made with the Image-Studio Lite 5.2.5 software, (LI-COR, NE, USA). **C)** Total protein extracts were immunoprecipitated (IP) with anti-NFAT or anti-MEF-2 antibodies as indicated. Western blot analysis of immunoprecipitated proteins was performed using antibody against MEF-2 or NFAT as indicated. The identity of IP proteins is as follows: Lane 1, Mock (Protein A Sepharose antibody-binding beads without antibody); Lane 2, NFAT IP; Lane 3 MEF-2 IP. (The data showed in the graphs can be found as supporting information in the S1 Dataset file. The complete image of the blot can be found in S3 Fig file).

It is known that interaction between NFAT and MEF-2 is important for the expression of the Nur77 and the β-MHC genes. [20, 27] Taking together, our overexpression and functional assays strongly suggest that an interaction between NFAT and MEF-2 is important for the regulation of *hCASQ2* gene transcription in neonatal cardiomyocytes. Hence, we evaluated whether both NFAT and MEF-2 are present within the proximal region of *CASQ2* gene promoter. To do so, we performed chromatin immunoprecipitation (ChIP) assays using specific antibodies against MEF-2c and NFATc3. The ChIP assays results showed MEF-2 and NFAT enrichment within the region between -259 to -21 bp of the proximal rat *CASQ2* promoter **(Fig 6A and 6B)**. To demonstrate a direct interaction between MEF-2 and NFAT, we performed protein co-immunoprecipitation. Our results showed that MEF-2c and NFATc3 proteins interact directly with each other **(Fig 6C)**. Taken together, these results demonstrate that NFAT interacts with MEF-2 bound to its -133 bp binding site at the *hCASQ2* gene promoter. In this way, NFAT cooperates with MEF-2 in the transcriptional activation of the *hCASQ2* gene.

**Fig 7:**
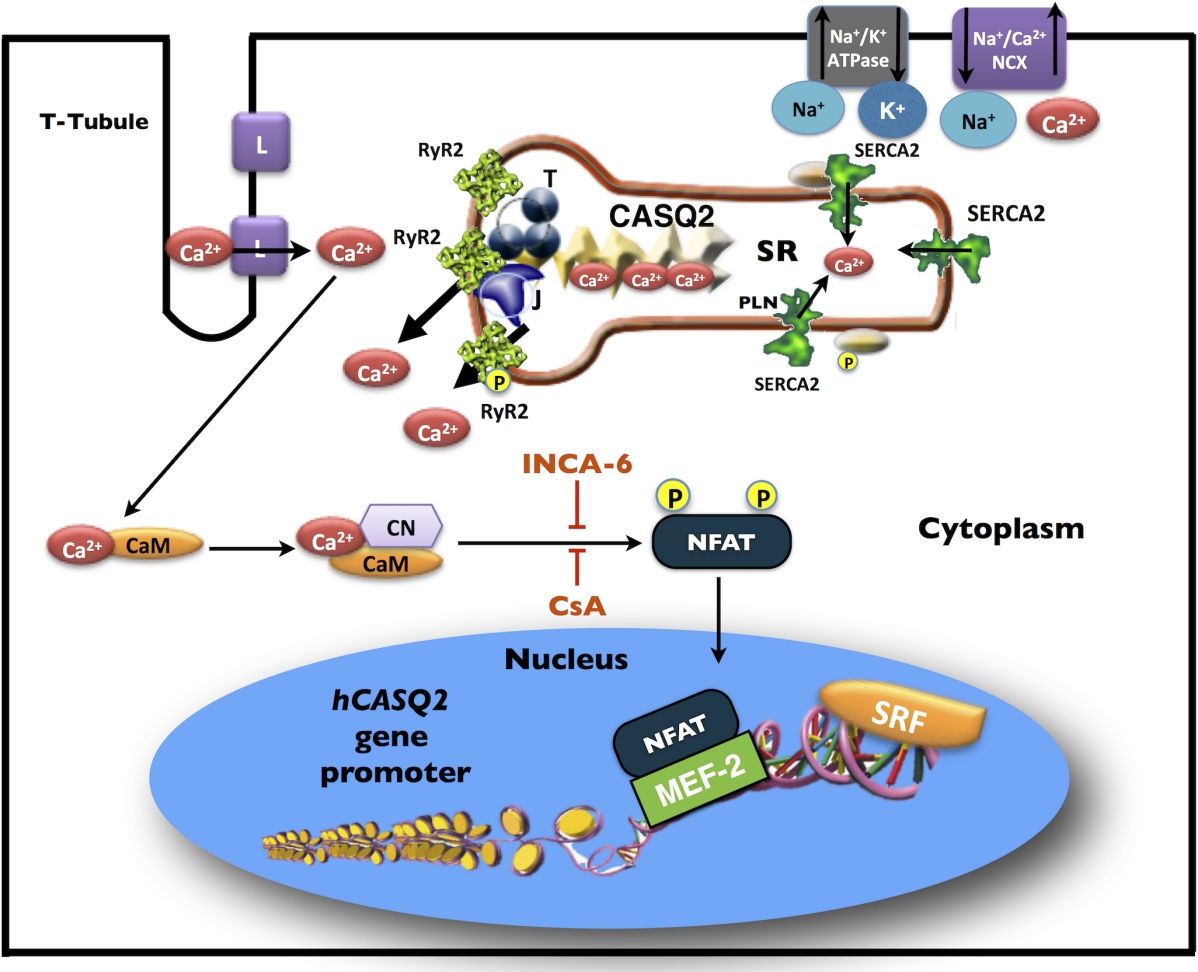
Proposed transcriptional regulation of *hCASQ2* gene in cardiomyocytes. CASQ2 is the main SR Ca^2+^-binding protein. In association with Triadin (T) and Junctin (J) proteins, CASQ2 is capable to regulate the activity of the SR Ca^2+^-release channel RyR2. NFAT transcription factor interacts with MEF-2 bound to its -133 bp binding site at the *hCASQ2* gene promoter. In this way, NFAT cooperates with MEF-2 in the transcriptional activation of the *hCASQ2* gene. Inhibition of NFAT activation with cyclosporine A (CsA) or INCA-6 blocks NFAT activation and down-regulates *hCASQ2* gene transcription. Abbreviations: CaM, calmodulin; CN, calcineurin; L, L-type Ca^2+^ channels; MEF-2, myocyte enhancer factor 2; NCX, Na^+^/Ca^2+^ exchanger; NFAT, nuclear factor of activated T cells; PLN, phospholamban; SRF, serum response factor; SR, sarcoplasmic reticulum.

In summary, we demonstrate that the inhibition of NFAT activation (with CsA or INCA-6) reduced the transcription of the *CASQ2* gene in primary cultures of neonatal rat cardiomyocytes. We also showed that overexpression of NFATc1 and NFATc3 in neonatal cardiomyocytes induced the transcriptional activity of the *hCASQ2* gene promoter constructs and that the mechanism responsible for this effect is explained by the interaction between MEF-2 and NFAT within the *hCASQ2* gene promoter **(Fig 7).**

## Discussion

In cardiomyocytes, CASQ2 is the most abundant protein inside the terminal cisternae of the SR. The main function of CASQ2 is to maintain the free SR Ca^2+^ concentration at 1 mM, but also has an important role in the regulation of SR Ca^2+^ release by the RyR2 Ca^2+^ channel in cardiomyocytes [10]. Mutations that insert premature stop codons or mutations that produce a nonfunctional CASQ2 protein result in an increased Ca^2+^ permeability of the RyR2 channel in resting conditions [13]. Phenotypically, this produces the development of ventricular arrhythmias such the cardiac polymorphic ventricular tachycardia [34]. In contrast, transgenic mice that overexpress CASQ2 develop cardiac hypertrophy [35]. CASQ2 overexpression induces a fetal gene expression program that results in impairment of contractile function with an increased Ca^2+^ content inside the SR but a reduced release of Ca^2+^ during the cardiac excitation-contraction coupling [35, 36]. CASQ2 overexpressing transgenic mice develop dilated cardiomyopathy with reduced SERCA2a expression and increased expression of cardiac remodeling-related proteins such cardiac ankyrin repeat protein (CARP), glutathione peroxidase, decorine (Dcn), TGF-b1-stimulated clone-36 protein (TSC-36), microfibril-associated glycoprotein-2 (Magp2) and osteoblast-specific factor-2 (Osf2) [37].

Despite the importance of CASQ2 protein in cardiac physiology, the regulation of the *CASQ2* gene expression is poorly understood. Previously, we have demonstrated that MEF-2 and SRF transcription factors bind to the promoter region of the human *CASQ2* gene and are important for its transcriptional regulation [16]. MEF-2 and SRF belong to the MADS-box family of proteins (MCM1, *Agamous*, *Deficiens*, SRF). These transcription factors also regulate the expression of many muscle-specific genes such as the α-MHC, MCK, myogenin, MyoD, MLCv2 and skeletal α-actin [24].

Besides MEF-2 and SRF, other transcription factors could regulate the expression of *CASQ2* gene. In this work, we investigated the role of NFAT in the regulation of the *hCASQ2* gene expression in neonatal rat cardiomyocytes. The NFAT transcription factors are related to the Rel/NFκB transcription factors family. The NFAT1-4 isoforms are expressed in cardiomyocytes, and their activity can be regulated by the action of Ca^2+^/CaM-dependent phosphatase calcineurin [19, 38]. Phosphorylated NFAT is located in the cytoplasm. When the cytoplasmic [Ca^2+^] is increased, Ca^2+^/CaM forms a complex with calcineurin, resulting in calcineurin activation and dephosphorylation of NFAT. Dephosphorylated NFAT is translocated into the nucleus where can activate its target genes [38]. The inhibition of calcineurin/NFAT pathway, with CsA, in Egr-1 overexpressing H9c2 cells, decreased the expression of rat CASQ2. This reduced expression of rat CASQ2 was mediated by the Egr-1 binding to the rat CASQ2 gene promoter [17]. As mentioned above, only the first 288 bp of the human *CASQ2* gene (*hCASQ2*) promoter have sequence homology between rat and human [16]. Because this region of the *hCASQ2* gene promoter lacks of an Egr-1 binding site, we searched for a mechanism independent of Egr-1.

Our results showed that the inhibition of NFAT activation reduced the transcription of CASQ2 mRNA in primary cultures of neonatal rat cardiomyocytes. In the same way, NFAT activation inhibition also reduced the transcriptional activity of *hCASQ2* gene promoter-chimeric constructs. *In silico* analysis of the *hCASQ2* gene promoter revealed a putative NFAT site located at position -230 bp. However, we did not observe any change in transcriptional activity of the putative -230 bp NFAT mutated construct compared to the wild-type construct. We hypothesized that NFAT interacts with MEF-2 bound to its -133 bp binding-site in *CASQ2* gene promoter. Our ChIP assays showed an enrichment of MEF-2 and NFAT within the region of -259 to -21 bp of the *hCASQ2* gene promoter. Previously, in Jurkat cells, it has been demonstrated that MEF-2 and NFAT regulate gene expression by a direct interaction between each other [26, 27]. The results of our protein co-immunoprecipitation assays, also demonstrated a direct interaction between MEF-2 and NFAT transcription factors in neonatal cardiomyocytes. Therefore, our results demonstrate that indeed, MEF-2 is directly bound to its binding site in the promoter (at -133 bp) and that NFAT is physically interacting with bound MEF-2. The above explain why the inhibition of NFAT activation reduced both the transcription of the endogenous *CASQ2* gene and the transcriptional activity of *hCASQ2* gene chimeric constructs. Thus, we demonstrated that the calcineurin/NFAT pathway regulates the transcription of the *hCASQ2* gene in neonatal cardiomyocytes. Then, as in the case of the β-MHC gene in skeletal muscle or the Nur77 gene in T-cells, the interaction between NFAT and MEF-2 is important for the regulation of *CASQ2* gene expression in cardiomyocytes [20, 27].

The activity of MEF-2 and NFAT transcription factors is increased in cardiac hypertrophy [39, 40]. It has been proposed that the interaction between MEF-2 and NFAT in cardiomyocytes promote the expression of genes associated with cardiac chambers dilatation [41]. As well, it is known that MEF-2 and NFAT are involved in the expression of heart failure markers ANP and BNP, respectively [42, 43]. Although the total levels of CASQ2 do not appear to be altered in cardiac hypertrophy or heart failure, alterations in the post-translational glycosylation or phosphorylation of CASQ2 protein were observed in a heart failure model [44–48]. This could change the normal targeting of CASQ2 protein into the SR and have an impact on the Ca^2+^ homeostasis of the cardiomyocytes [48].

Besides of calcineurin/NFAT pathway, other mechanisms cooperate to regulate the expression of the *hCASQ2* gene in cardiomyocytes. For instance, oxidative stress could have an impact on the regulation of *CASQ2* gene expression. Primary cultures of rat cardiomyocytes treated with FCCP (carbonyl cyanide-4-(trifluoromethoxy)-phenylhydrazone) showed a reduced level of CASQ2 mRNA, which could be prevented by treatment with the antioxidant agent N-acetylcysteine [49]. Moreover, mice treated in utero with diethylstilbestrol showed up-regulation of SR proteins involved in Ca^2+^-homeostasis control, such SERCA2a, NCX1, and CASQ2 [50]. This *CASQ2* gene up-regulation was associated with an increased methylation of *CASQ2* gene promoter [50]. Also, treatment of H9c2 cells with the methylation inhibitor 5-aza-deoxycytidine reduced *CASQ2* gene expression [17]. These results suggest that epigenetic mechanisms could have a role in the regulation of *CASQ2* gene expression. However, the molecular mechanism responsible for these effects must be investigated in greater detail [17, 50].

In conclusion, we demonstrate that the calcineurin/NFAT pathway regulates the *hCASQ2* gene transcription in neonatal cardiomyocytes through an interaction between MEF-2 and NFAT transcription factors at the *hCASQ2* gene promoter.

## Supporting information

S1 Dataset Dataset from the graphs. (ZIP)

S1 Fig Complete and unadjusted image of blot from Fig 1. (TIFF)

S2 Fig Complete and unadjusted image of blot from Fig 3. (TIFF)

S3 Fig Complete and unadjusted image of blot from Fig 6. (TIFF)

S1 Table Oligonucleotides for real-time qPCR, mutagenesis, and ChIP. (DOCX)
