# Supplementary material for: The cardiac calsequestrin gene transcription is modulated at the promoter by NFAT and MEF-2 transcription factors
Source: PLoS One. 2017 Sep 8;12(9):e0184724. doi: 10.1371/journal.pone.0184724 (PMC5590987; doi:10.1371/journal.pone.0184724)
Supplement: S1 Dataset — (ZIP) [file pone.0184724.s001.zip › S1_Table.docx]

**S1 Table.** **Oligonucleotides for real-time qPCR, Mutagenesis, and ChIP.**

| qPCR | CASQ2 Forward: 5´-AGCTTGTGGAGTTTGTGAAG-3´  CASQ2 Reverse: 5´-GGATTGTCAGTGTTGTCCC-3´  GAPDH Forward: 5´-GGAGAAACCTGCCAAGTATGATGAC-3´  GAPDH Reverse: 5´-TGGGAGTTGCTGTTGAAGTCG-3´ |
| --- | --- |
| Mutagenesis | NFAT -230 bp Mut Forward:  5´-ATTCTTGTCTTGGGCCTTCTGCCTCTTCCCTCTTCACCAGTCGGACAGAC-3´  NFAT -230 bp Mut Reverse:  5´-GAAGAGGCAGAAGGCCCAAGACAAGAATGTACACGCATGAAATAAGGAGG-3´  MEF-2 -133 bp Mut Forward:  5´-CATTGCATGCTGGATCCTCCGCCTGAAACAAC-3´  MEF-2 -133 bp Mut Reverse:  5´-GTTGTTTCAGGCGGAGGATCCAGCATGCAATG-3´  SRF -103 bp Mut Forward:  5´-CTGCATCCTAAAAATTTAGTTCC-3´  SRF -103 bp Mut Reverse:  5´-GGAACTAAATTTTTAGGATGCAG-3´ |
| ChIP | Forward: 5´-TGCCCCTTTATTTCACTTGC-3´  Reverse: 5´-GGAGGCCAAGCCCTAGATAC-3´ |
