# Supplementary figures and images for: The cardiac calsequestrin gene transcription is modulated at the promoter by NFAT and MEF-2 transcription factors
Source: PLoS One. 2017 Sep 8;12(9):e0184724. doi: 10.1371/journal.pone.0184724 (PMC5590987; doi:10.1371/journal.pone.0184724)

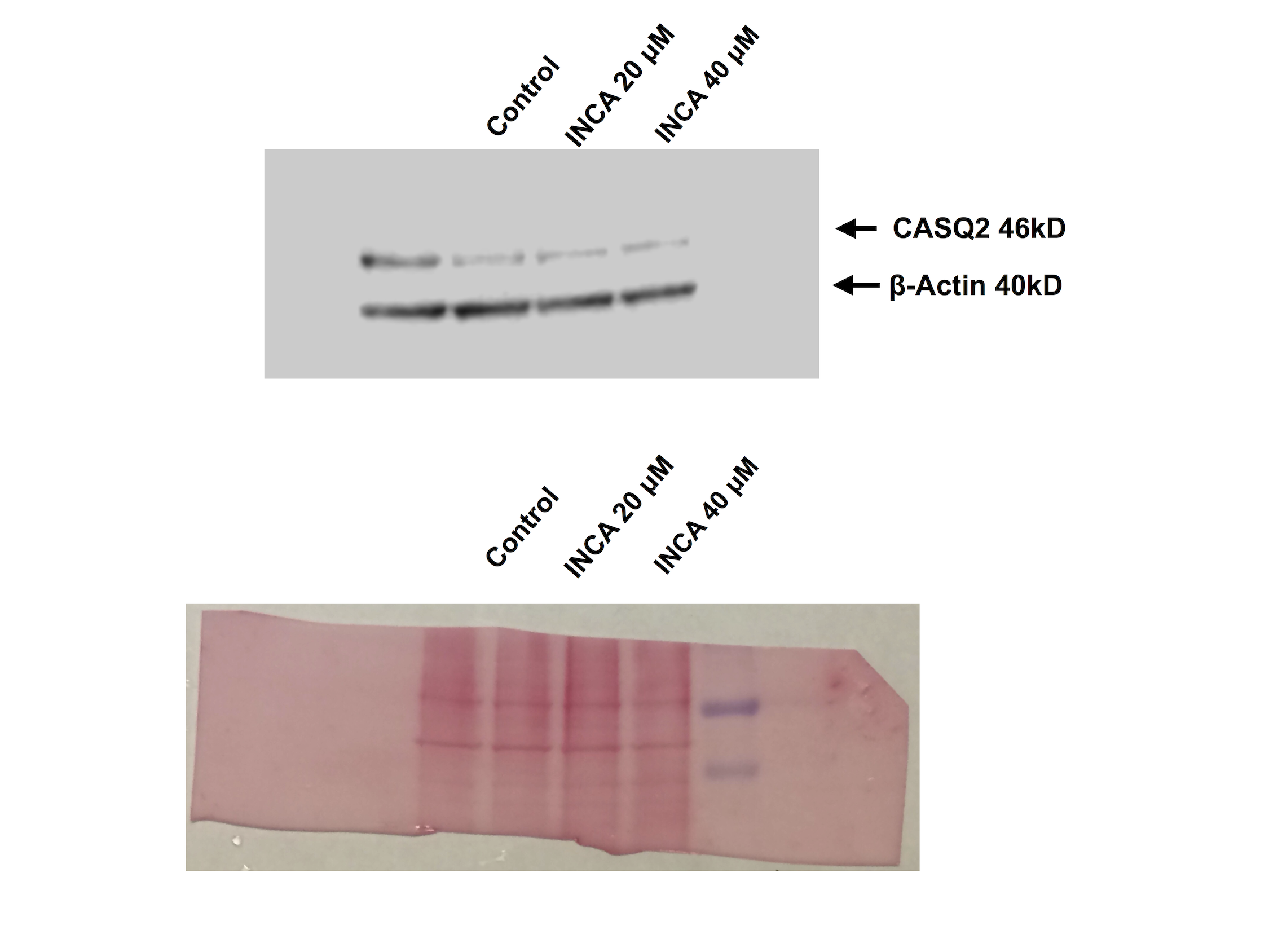

Supplement: S1 Dataset — (ZIP) [file pone.0184724.s001.zip › S1_Fig.tiff]

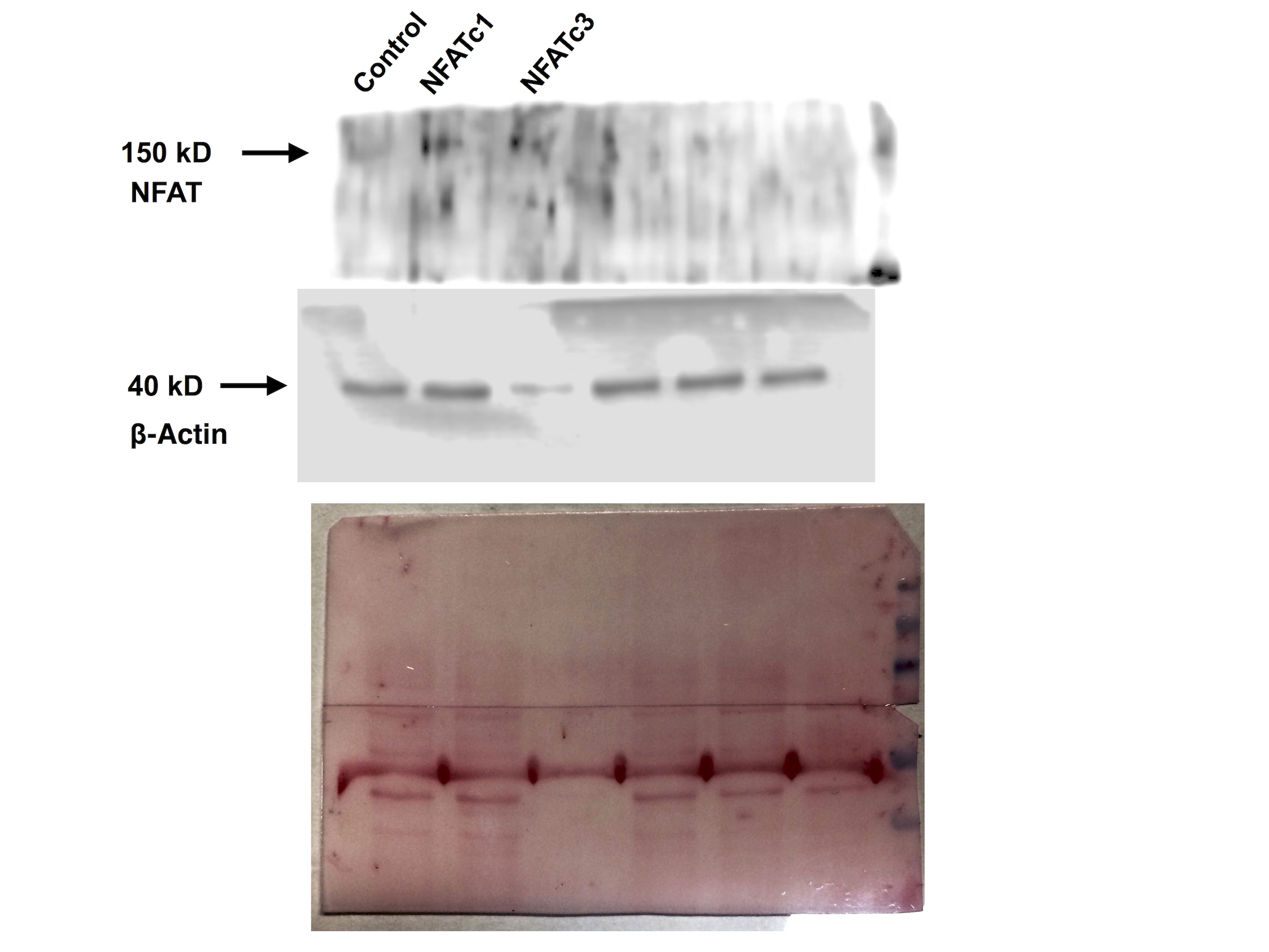

Supplement: S1 Dataset — (ZIP) [file pone.0184724.s001.zip › S2_Fig.tiff]

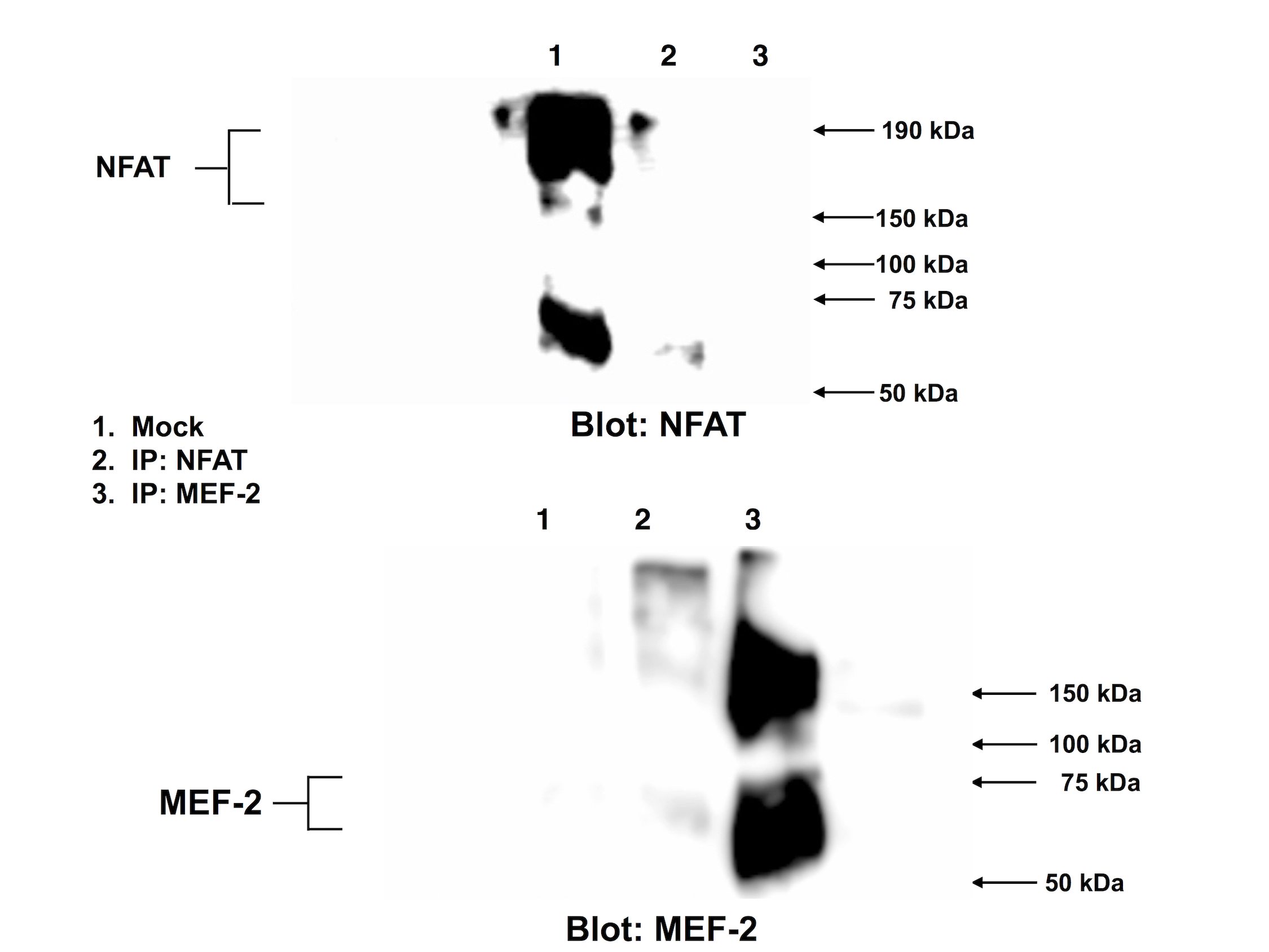

Supplement: S1 Dataset — (ZIP) [file pone.0184724.s001.zip › S3_Fig.tiff]
